# Management of Surgical Margins in the Excision of Oral Leukoplakia: A Scoping Review

**DOI:** 10.1111/odi.70231

**Published:** 2026-02-13

**Authors:** Mateus José Dutra, Isaac Santos Araujo, Vivian Petersen Wagner, Isabel Schausltz Pereira Faustino, Marco Meleti, Nikolaos Nikitakis, Márcio Ajudarte Lopes, Alan Roger Santos‐Silva, Caique Mariano Pedroso

**Affiliations:** ^1^ Department of Oral Diagnosis Piracicaba Dental School, Universidade Estadual de Campinas Piracicaba SP Brazil; ^2^ Department of Oral Medicine, School of Dentistry Universidade de São Paulo São Paulo SP Brazil; ^3^ Department of Stomatology, Public Health and Forensic Dentistry, Ribeirão Preto Dental School Universidade de São Paulo Ribeirão Preto SP Brazil; ^4^ Department of Surgery and Medicine Università di Parma Parma Italy; ^5^ Department of Oral Medicine & Pathology and Hospital Dentistry, School of Dentistry, National and Kapodistrian University of Athens Athens Greece

**Keywords:** clinical protocols, laser therapy, margins of excision, oral leukoplakia

## Abstract

**Objective:**

To map the literature on management of surgical margins for oral leukoplakia (OL) and proliferative verrucous leukoplakia (PVL).

**Methods:**

Searches were performed in five electronic databases and gray literature. Studies involving patients undergoing any type of surgical excision of OL or PVL were included. Data extraction focused on surgical technique, margin size, method of margin delineation, and recurrence outcome. Margin size was categorized in centimeters.

**Results:**

Ninety‐one studies were included. More than half of the studies (54.9%) did not report the width of surgical margins. Among those that did, margins between > 2 and < 3 mm were the most frequently reported (19.7%). Notably, 94.5% of the studies did not specify the criteria used to determine the margin width. Furthermore, 76.9% failed to describe the method used to delineate lesion margins. High recurrence was observed in 15.1% of OL cases with surgical margins ≤ 2 mm and in 40% of PVL cases with margins > 2 to ≤ 3 mm. Lesions with margins between 3 and 5 mm showed a lower recurrence rate.

**Conclusion:**

We identified a lack of standardization in the reporting of surgical margins. Excision with 5 mm of clinically healthy tissue should be considered the optimal approach for the surgical management.

## 1 Introduction

Oral potentially malignant disorders (OPMD) are considered a diverse group of mucosal lesions associated with an increased risk of progression to oral cancer (Warnakulasuriya et al. 2021). Oral leukoplakia (OL) is the most prevalent OPMD in clinical practice, of which the malignant transformation rate varies according to the population and associated risk factors, with a rate of 13.07% (Saldivia‐Siracusa and González‐Arriagada 2021). A particularly aggressive subtype of OL is proliferative verrucous leukoplakia (PVL), distinguished by its multifocal presentation, progressive course, high recurrence, and elevated risk of malignant transformation (Warnakulasuriya et al. 2021; Hansen et al. 1985; Warnakulasuriya 2018). PVL often mimics other OPMD in its early stages, such as lichen planus, complicating its clinical diagnosis (Lopes et al. 2015; Dutra et al. 2023). Despite its aggressive nature, the literature suggests that oral squamous cell carcinoma (OSCC) originating from PVL might exhibit a more favorable prognosis than conventional OSCC (Faustino et al. 2023).

Several treatment modalities have been explored for the treatment of OL and PVL, including topical or systemic retinoids (Matulić et al. 2019), cryotherapy (Pandey et al. 2023), chemoprevention agents (Dutra et al. 2024), and various surgical approaches. Surgical excision remains the first choice of treatment for both OL and PVL, aiming to reduce the risk of recurrence and malignant transformation (de Pauli Paglioni et al. 2024). Although surgical removal may reduce the chances of malignant transformation (van der Waal 2009; Mehanna et al. 2009), this risk is not entirely eliminated (Warnakulasuriya and Ariyawardana 2016). Excisional techniques range from conventional scalpel to different types of surgical lasers (Monteiro et al. 2017; de Pauli Paglioni et al. 2024). However, the diversity of laser types, device parameters, and surgical techniques presents challenges in standardizing treatment protocols. Even conventional scalpel excision varies substantially across clinical settings. In this context, the lack of uniformity in surgical protocols, particularly regarding the selection and management of surgical margins, can impact treatment outcomes.

In oncology setting, a 5 mm margin of healthy tissue around the lesion is considered the standard in oral cavity cancer (Robbins et al. 2019; Li et al. 2019). However, standard surgical margins for surgical management still inconsistent for OL and PVL. Inconsistent reporting and methodological variability across studies limit the reproducibility and comparability findings, and may inadvertently contribute to recurrence or malignant progression (Pedroso et al. 2024). Therefore, the aim of this scoping review was to systematically map the available evidence from primary studies on surgical excision techniques for OL and PVL, with particular emphasis on how clinical surgical margins are selected and managed. By identifying methodological patterns and gaps, this review objective informs future research and contributes to the development of more standardized and effective surgical protocols.

## 2 Methods

### 2.1 Protocol and Registration

This scoping review was conducted following the Joanna Briggs Institute principles and the Preferred Reporting Items for Systematic Reviews and Meta‐Analyses for Scoping Reviews (PRISMA‐ScR) extension guidelines (Tricco et al. 2018). The protocol was registered in the Open Science Framework (https://osf.io/6pxya).

### 2.2 Eligibility Criteria

#### 2.2.1 Inclusion Criteria

##### 2.2.1.1 Population

We included studies involving patients diagnosed with either OL or PVL. OL was defined as a predominantly white plaque of questionable risk that cannot be characterized clinically or histopathologically as any other definable disease (Warnakulasuriya et al. 2021). PVL was defined as a progressive, persistent, and irreversible disorder, characterized by the presence of multiple OL that frequently become verrucous over time and a high‐risk of malignant transformation (Warnakulasuriya et al. 2021).

##### 2.2.1.2 Context

Eligible studies were those reporting on surgical treatment modalities for OL and PVL. This included any form of surgical excision such as laser surgery, electrocautery, cryosurgery, or conventional scalpel excision (Starzyńska et al. 2015; de Pauli Paglioni et al. 2024).

##### 2.2.1.3 Type of Evidence

We considered primary clinical evidence from observational (cross‐sectional, case–control, or cohort studies) or interventional studies (randomized or nonrandomized controlled trial) without restriction on publication year or language.

#### 2.2.2 Exclusion Criteria

We excluded studies that met any of the following criteria: (a) Full text or essential data unavailable, (b) Data extraction was impossible, (c) Secondary literature (literature reviews, letters to the editor, short communications, personal opinions, study records, book chapters or conference abstracts), (d) Case reports or case series, (e) In vitro or animal studies, (f) Epidemiological studies without surgical focus, (g) Studies centered on photodynamic therapy, diagnostic techniques, nonsurgical, or topical treatments, (h) Immunohistochemical and molecular marker studies not focused on surgical intervention.

### 2.3 Information Sources and Search

We conducted systematic searches in the following databases: Scopus, PubMed, Embase, Web of Science, and Cochrane Library. To enhance the scope of the search, gray literature was explored through Google Scholar and ProQuest Dissertations and Theses. Also, the reference lists of all included studies were manually screened to identify potentially relevant publications that may have been missed during the electronic search. All database investigations were conducted on December 27th, 2024. The electronic search strategy was adapted in each database (Table S1) according to the following keywords: “Leukoplakia,” “Therapeutics,” “Laser Therapy,” “Electrocoagulation,” “Electrocautery,” and “Cryo Therapies.”

### 2.4 Selection of Source of Evidence

After database screening, we performed a duplicate removal two‐step procedure. First, one author (M.J.D.) exported records from each database to the EndNote software (Clarivate Analytics, Philadelphia, PA, USA) and removed duplicates. The same author subsequently imported the remaining records to the online software Rayyan (Rayyan, Qatar Computing Research Institute) and manually removed residual duplicates. After deleting duplicates, we applied a two‐phase process to select the final studies. First, two authors (M.J.D. and C.M.P.) selected the records by titles and abstracts, subsequently, selected studies with appropriate titles and abstracts were directed to the next phase to review the full texts. Disagreements were resolved by discussion and consultation with a third author (A.R.S.‐S.).

### 2.5 Data Chart Process

A data extraction sheet adapted or guided by to the scope of this review was created to capture the following data fields: (a) study characteristics: author(s), year of publication, country, type of study; (b) patient and lesion details: clinical diagnosis (OL or PVL), patient sex, mean age, degree of epithelial dysplasia (no dysplasia, mild, moderate, or severe) and tobacco and alcohol consumption; (c) surgical information: type of excision, procedure modality, surgical tools, clinical margins (quantity included, in millimeters, of healthy tissue), and references used to justify margin width; (d) outcomes: mean follow‐up period (in months) and the number of recurrences. The data were extracted by one author (M.J.D.) and reviewed by another (C.M.P.); discrepancies were resolved by consulting a third author (A.R.S.‐S.). The data were tabulated in a table in Microsoft Excel software, for later overview of the data and creation of tables presenting the results.

### 2.6 Data Items

Surgical margins were defined as the anatomical zone where the incision is made to completely remove the lesion. In cases of surgical excision, margins refer to the distance from the visible (peripheral or deep) edge of the lesion to the incision site.

### 2.7 Synthesis of Results

The extracted data were synthesized using a descriptive approach. Findings were summarized in tables reporting frequency, percentage, mean, and, where applicable, minimum and maximum values. Surgical margins were classified based on the definitions provided in each study or inferred when ranges were reported. The categorization was as follows: (a) Not Reported (NR): Studies that did not specify the margin width; (b) ≤ 2 mm: margins defined as 0–2 mm, which may reflect minimal or questionable margins; (c) > 2 to ≤ 3 mm: defined as narrow margins, typically 2–3 mm; (d) > 3 to ≤ 5 mm: considered a standard “safe” margin, typically ranging from 3 to 5 mm; (e) > 5 mm: margins reported as 6 mm, within the range of 3–6 or 10 mm, indicating more aggressive excisions; and (f) ranged: Studies that presented a range of margin widths (e.g., 0–5, 1–3, 2–5 mm) without a fixed or mean value were grouped separately.

## 3 Results

### 3.1 Study Selection

The database searches identified 1.030 records. After removing duplicates, 586 articles were selected for title and abstract screening. Of these, 230 were selected for full‐text review resulting in 84 articles included for data extraction. Considering the gray literature, seven additional articles were included. Overall, 91 studies were included in this review. The search process results are illustrated in the PRISMA flowchart (Figure S1).

### 3.2 Characteristics of Source of Evidence

The countries with the highest number of publications were India, Italy, and Taiwan, accounting for 13.1%, 10.9%, and 9.8%, respectively (Table S2). The predominant study types were retrospective studies (58.2%), prospective studies (15.3%), and comparative studies (9.8%). Regarding diagnosis, 9.082 cases were of OL (98.2%) and 163 cases of PVL (1.76%). Regarding the histology of the 9082 cases of OL, most did not present epithelial dysplasia (43%). Mild dysplasia was observed in 10.6% of cases, moderate in 4.09%, and severe in 2.73%, while 39.5% of the cases did not have this data reported or assessed. For the 163 cases of PVL, 14.1% did not present dysplasia in the histological evaluation, 28.8% presented mild dysplasia, 11% moderate dysplasia, and 3.68% severe dysplasia, while approximately 42% of the cases did not have this information assessed or reported by the primary studies (Table S2). The overall mean age was 57.2 years. The mean follow‐up duration was 29.4 months (Table S2).

Several surgical tools were reported for OL excision. The most common surgical tool was CO_2_ laser (73.9%). The traditional scalpel was used in 7.8% of cases, followed by cryosurgery (4.9%), Er:YAG laser (3.9%) and diode laser (3.6%). The most frequently employed treatment technique was excision combined with vaporization, ablation, or coagulation (41.7%), followed by excision alone (35%) and vaporization alone (10.5%). Other combined techniques were used in smaller percentages (Table S3).

For PVL, CO_2_ laser was also the most frequently used surgical tool (71.7% of cases), followed by the traditional scalpel (14.7%). The most commonly employed technique for PVL was excision combined with vaporization, ablation, or coagulation (62.5%). Vaporization alone was used in 11.6% of cases (Table S3).

Of the 9082 cases of OL, 1030 (11.3%) experienced recurrence. Among the 163 cases of PVL, 34 (20.8%) recurred. For both lesion types, the highest recurrence rates were observed in lesions treated with CO_2_ laser: 28.2% in PVL cases and 30.6% in OL cases (Table S3).

### 3.3 Synthesis of Results

Only 45.1% (*n* = 41) of the studies reported the inclusion of healthy tissue in the margins of excised lesions. Among these studies, the margin ranged from 0 to 10 mm. Eighteen (19.7%) studies included > 2 to ≤ 3 mm of healthy tissue in the surgical margins, 11 (12%) included > 3 to ≤ 5 mm, 4 (4.3%) included ≤ 2 mm, and 2 (2.1%) > 5 mm. Another six studies (6.5%) included measurements that could range from 0 to 5 mm (Table 1).

**TABLE 1:** Information on surgical margin management, considering whether healthy tissue was included and how many millimeters.

Surgical margin (healthy tissue included)	Definition	*N* (studies)	%	Rationale
NR	Not reported	50	54.9	Studies did not provide margin width
≤ 2 mm	0–2 mm	4	4.3	May reflect minimal or questionable margins
> 2 to ≤ 3 mm	2–3 mm	18	19.7	Narrow margin
> 3 to ≤ 5 mm	3–5 mm	11	12	Standard “safe” margin
> 5 mm	6, 3–6, or 10 mm	2	2.1	Rare and more aggressive excisions
Ranged	0–5, 0–3, 1–3, 1–5, 2–5 mm	6	6.5	Studies reported broad range without fixed margin

Abbreviations: mm, millimeters; *N*, number; NR, not reported.

Considering whether the methodologies followed any bibliographic reference to define the number of millimeters of healthy tissue to be included in the margins, only 5 (5.1%) studies cited references (Table 2). Regarding the method of defining the lesion margins, 70 articles (76.9%) did not describe how this was determined. Margin delineation by laser pulse or continuous mode was used in seven studies (7.6%), toluidine blue staining in 4 (4.3%), and the use of demographic/surgical marker pens was reported in four studies (4.3%) as methods for margin delimitation (Table 2).

**TABLE 2:** Information on the definition of surgical margins, considering how the studies defined them and bibliographic references used.

Surgical margins delimitation	*N (studies)*	%
NR	70	76.9
Laser pulse or continuous mode outlining	7	7.6
Toluidine blue staining	4	4.3
Dermographic‐surgical marker pen	4	4.3
Lugol's iodine staining	2	2.1
Ink marking	2	2.1
Methylene blue marking	1	1
Moat incision around the lesion	1	1
Total	91	100
Reference for defining margins (delimitation and millimeters)		
NR	86	94.5
Vedtofte et al. (1987)	2	2.1
Lingen et al. (2017)	1	1
Nammour et al. (2017)	1	1
Panje et al. (1989)	1	1

Abbreviations: *N*, number; NR, not reported.

In cases of OL, the highest recurrence rate (15.1%) was observed when ≤ 2 mm of healthy tissue was included in the surgical margins (Table 3). For PVL, recurrence was 40% when margins included > 2 to ≤ 3 mm of healthy tissue and 21.3% when the extent of healthy tissue was not reported or could not be determined from the studies (Table 3).

**TABLE 3:** Information on cases of leukoplakia and proliferative verrucous leukoplakia according to the amount of healthy tissue included in the surgical margin and their respective recurrences.

Surgical margin	Margin details	OL treated *N* (%)	OL recurrence *N* (%)	PVL treated *N* (%)	PVL recurrence *N* (%)
NR	NR	3.274 (100)	476 (14.5)	150 (100)	32 (21.3)
≤ 2 mm	0–2 mm	139 (100)	21 (15.1)	—	—
> 2 to ≤ 3 mm	2–3 mm	2.246 (100)	327 (14.5)	5 (100)	2 (40)
> 3 to ≤ 5 mm	3–5 mm	703 (100)	85 (12.09)	8 (100)	—
> 5 mm	6, 3–6, or 10 mm	67 (100)	5 (7.46)	—	—
Ranged	0–5, 0–3, 1–3, 1–5, 2–5 mm	2.653 (100)	116 (4.37)	—	—
Total		9.082 (100)	1.030 (11.3)	163 (100)	34 (20.8)

Abbreviations: mm, millimeters; NR, not reported; OL, oral leukoplakia; PVL, proliferative verrucous leukoplakia.

## 4 Discussion

There is a lack of standardization in the selection and management of surgical margins for the treatment of OL and PVL. Our review revealed considerable variability in the margins employed for excision, both in absolute measurements and in how they were defined, ranging from fixed values to broad, unspecified ranges. Notably, more than half of the studies (54.9%) did not report the surgical margins used, underscoring a significant methodological gap. This lack of consistency may hinder reproducibility and influence clinical outcomes, particularly recurrence rates. As previously noted by Pedroso et al. (2024), surgical protocols for dysplastic lesions are diverse, and our findings confirm that such heterogeneity also applies to OL and PVL management.

Our search revealed a wide range of surgical protocols for the excisional treatment of OL, and to a lesser extent, PVL. A variety of instruments were employed for lesion removal, including conventional scalpels, cryosurgery, and different types of surgical lasers. These methods differed not only in technique (excision, ablation or vaporization) but also in the definition and management of surgical margins, which directly impacted clinical outcomes. The approaches described ranged from complete excision (entire lesion removal) to ablation (deeper tissue removal) and vaporization (more superficial tissue removal). Pedroso et al. (2024) emphasized the difficulty in defining an optimal surgical approach for dysplastic lesions. Our results align with theirs. Even among studies referencing established criteria, substantial inconsistencies were found regarding the extent of clinically healthy tissue and how margins were demarcated. In line with this perception, Araújo et al. (2023), indicated that clinicians interpret OPMD differently regarding lesion delimitation. In this context, clinical subjectivity in delimiting where the lesion begins and ends may be a crucial aspect for determining the surgical margin.

Only five studies cited specific references for margin management, underscoring a significant lack of standardization in this procedure. Two studies followed the criteria described by Vedtofte et al. (1987), who marked lesions with ink and included 3–5 mm of clinically healthy mucosa in the excision of 61 patients. Another study referenced Lingen et al. (2017); however, we were unable to identify any margin management criteria for OPMD in that publication. A fourth study cited Nammour et al. (2017), in which 2347 cases of OL were treated with margins of 1–3 mm of healthy tissue. The fifth study referred to Panje et al. (1989), who treated 71 patients with malignant and OPMDs using laser excision, incorporating a rim of clinically healthy tissue at the resection edges. Overall, even among studies that cite reference criteria, detailed procedural descriptions are often lacking, with some failing to specify the width of surgical margins. This inconsistency, combined with small sample sizes, may result in suboptimal clinical decision‐making and potential errors in determining appropriate surgical margins.

Based on our review, the most consistent and effective approach to margin management involves clinically delineating the lesion using ink or a surgical marker and excising 3–6 mm of surrounding clinically healthy tissue while accounting for anatomical constraints. This method, also supported by Nammour et al. (2017), may help reduce recurrence by promoting complete lesion removal. Recurrence after excision is multifactorial. Although tobacco and alcohol use are well‐established risk factors (Chandu and Smith 2005; Yang et al. 2011), only 5.8% and 13.5% of patients in our dataset were documented as alcohol and tobacco users, respectively. These figures likely underestimate true prevalence due to underreporting, representing a limitation of our analysis. Surgical margin management remains a key determinant of outcomes. Minimal resection, positive margins, or incomplete excision substantially increase the risk of recurrence and malignant transformation (Ballivet de Régloix et al. 2018). Our findings corroborate this: studies using narrow margins (0–3 mm) or employing lesion ablation/vaporization rather than complete excision reported higher recurrence rates. These techniques may leave residual dysplastic tissue, thereby increasing the risk of recurrence (Brouns et al. 2013; Saibene et al. 2019).

Our findings suggest a potential association between narrower surgical margins and higher recurrence rates in both OL and PVL, highlighting the critical role of margin width in treatment outcomes. Specifically, OL cases with ≤ 2 mm margins showed the highest recurrence rate (15.1%), while those with > 5 mm margins had the lowest (7.46%). Similarly, PVL cases with > 2 to ≤ 3 mm margins had a markedly elevated recurrence rate (40%) compared to cases with unreported margin width (21.3%). Moreover, the aggressive and multifocal nature of PVL, often associated with high recurrence and malignant transformation rates (Warnakulasuriya 2018; Proaño‐Haro et al. 2021), reinforces the need for wider excisions and vigilant follow‐up. Notably, in our analysis, a large proportion of OL cases fell within the “ranged” category (e.g., 0–5 mm), showing relatively low recurrence (4.37%), which may reflect variability in how surgical margins are reported or interpreted across studies. These results underscore the importance of consistent documentation of surgical margin widths and the consideration of wider excisions, particularly in high‐risk lesions such as PVL. Despite this, it is important to mention that recurrence after different treatments may be related to other factors, such as alcohol and tobacco consumption, which were observed in 5.8% and 13.5% of the cases included in this review, respectively, as well as the histological grade, where different levels of epithelial dysplasia were reported in 17.4% of OL cases and 43.4% of PVL cases.

We must consider that, in some areas of the oral cavity, the anatomy does not favor the ideal delimitation of surgical margins in extension and depth, which may have caused the variability observed in these studies. In such cases, surveillance should be more rigorous, or complementary treatments after surgery may be employed, such as the use of vitamin A or beta‐carotene, which may be effective in reducing OL, although they do not prevent cancer development in individuals with this OPMD (Lodi et al. 2016). In addition, other methods include peri‐incisional CO_2_ laser vaporization of the surrounding keratinized mucosa after scalpel excision to treat subclinical field alterations (Ishii et al. 2003); photodynamic therapy (e.g., 5‐ALA‐based) targeted at the resection bed and adjacent mucosa to treat residual dysplastic foci (Ou et al. 2022); cryotherapy applied to the defect margin for ablation of microscopic disease (Pandey et al. 2023); short courses of topical chemopreventive agents, such as imiquimod, on residual or satellite plaques (Sroussi et al. 2025); and systemic retinoids as a postoperative chemopreventive strategy, as well as lifestyle modifications (smoking and alcohol cessation), which are also commonly included in the adjuvant plan following surgical removal (Lodi et al. 2016).

Several limitations of this review should be acknowledged. The inability to extract relevant data, particularly regarding margin width, from many studies limited the possibility of more consistent analyses. In addition, only a small number of studies reported outcomes for PVL, making direct comparisons with OL challenging. Furthermore, the recurrence data we reported were based on the available follow‐up information, which varied widely across studies, therefore, recurrence rate estimates should be interpreted with caution. Finally, the recurrences observed may have been influenced by other factors such as tobacco use, alcohol consumption, and patterns of epithelial dysplasia, however, such associations cannot be confirmed, as information on these factors was available for only a minority of cases. These limitations delineate clear evidence gaps and should help guide future, adequately powered clinical studies aimed at refining margin assessment and improving surgical protocols for the management of these patients.

### 4.1 PVL Considerations

Some important considerations must be raised regarding PVL, which curiously presents peculiar characteristics. This OPMD occurs mainly in women, without associated risk factors such as tobacco and alcohol use, and is also characterized by a progressive clinical course (Staines and Rogers 2024). Regarding diagnosis, the tools that assist in this process, such as chemiluminescence or autofluorescence, have low specificity, limiting the clinical utility of these adjuncts for the diagnosis and monitoring of PVL (Rashid and Warnakulasuriya 2015). Moreover, recurrences are common regardless of the surgical or adjuvant technique employed, therefore, patients with PVL require strict, long‐term surveillance, and risk factors must be eliminated, especially tobacco and alcohol consumption (Capella et al. 2017; Proaño‐Haro et al. 2021). By its nature, as it is refractory to available treatments, PVL is also characterized by high rates of malignant transformation (Capella et al. 2017; Proaño‐Haro et al. 2021). In this context, PVL has its singular features and, therefore, even with its high recurrence rates after different treatments, seemingly more related to the pathogenesis of this OPMD, we must also consider and rule out possibilities related to excisional treatments, particularly regarding the persistence of residual disease in compromised margins, which may influence better outcomes. Therefore, when excision is chosen as the treatment method, we recommend including at least 3 mm of healthy tissue in the surgical margins of PVL, ideally 5 mm, considering the anatomical limitations of each case.

### 4.2 Future Directions and Recommendations

Given the significant heterogeneity and lack of standardization in the management of surgical margins observed in this review, there is an urgent need for the development of evidence‐based clinical guidelines to assist professionals in defining adequate margins for the excision of OL and PVL. Based on our analysis of more than 9000 cases of OL and 163 cases of PVL, we recommend that lesions be clinically delineated using ink or surgical markers, and propose that excision margins include at least 5 mm of clinically healthy tissue beyond the visible lesion. This approach is supported by lower recurrence rates in studies that use margins within this range and is further aligned with the findings of Nammour et al. (2017), which suggest better outcomes with wider margins. Moreover, we recommend that future studies consistently report surgical margin width, the method of delineation, and histopathological confirmation of margin status, among other details, to enhance reproducibility and enable more robust comparisons across studies. In this context, we propose here the minimum requirements that should be reported in the methodology and results of studies addressing different surgical techniques for the excision of OPMDs (Table 4), and we encourage such criteria to be included in future studies to standardize reporting and facilitate future comparisons. Finally, prospective multicenter studies comparing different margin widths and excisional methods (scalpel vs. laser) in OL and PVL are essential to determine the most effective and safe approaches to reduce recurrence and malignant transformation.

**TABLE 4:** Proposal for minimum methodological reporting requirements for clinical studies on the surgical treatment of OPMDs.

1. Trial design and identification	Present the type and objectives of the study
2. Ethics and registration	Ethical issues and protocol records
3. Population and clinical data	Population data (origin, ethnicity, country, sex, age, tobacco and alcohol consumption, and exposure to other risk factors) and clinical information (location, number of lesions, time of evolution, size)
4. Clinical diagnosis	What was the clinical diagnosis, specifying which OPMD
5. Histopathological diagnosis	Histopathological diagnosis considering the presence or absence of dysplasia, and when present, the degree of dysplasia
6. Type of tool used in excision	Specify whether it was a conventional scalpel, different types of surgical laser (CO_2_, diode laser…), cryosurgery, or other means
7. Method used for removal	Surgical removal, ablation, photocoagulation, vaporization…
8. How the margins were defined	How was the area to be incised marked, and what tools were used for marking (surgical pen, surgical ink, or other methods…)
9. How many millimeters of healthy tissue were included in the margins from the clinical edge of the lesion	Number of millimeters of distance between the clinical edge of the lesion and the place where the incision was made
10. Complementary treatments at the surgical margin	Specify whether after excision any other approach was performed on the surgical margin (ablation, cryotherapy, coagulation…)
11. What study was used as a parameter to define the margin millimeters	Cite which study was used as a reference to support the amount of healthy tissue included in the surgical margins
12. Follow up time	Patient follow‐up period, in months or years, after excisional treatment
13. Recurrence	Information on recurrence after excisional treatment

Abbreviation: CO_2_, carbon laser.

## 5 Conclusion

This review highlights a significant lack of uniformity and transparency in the definition and reporting of surgical margins in the treatment of OL and PVL. More than half of the studies did not specify margin width. We suggest that the use of well‐defined clinical margins, combined with the inclusion of at least 5 mm of healthy tissue in the excision, may reduce recurrence rates, particularly when complete surgical excision is prioritized over ablative methods. Future research and clinical trials should prioritize standardized reporting of surgical parameters and rigorously evaluate the relationship between margin width and clinical outcomes to support the development of clear clinical guidelines and validate the recommendations proposed by our study.

## Author Contributions

**Mateus José Dutra:** investigation, writing – original draft, methodology, validation, visualization, writing – review and editing, data curation. **Isaac Santos Araujo:** validation, visualization, writing – review and editing. **Vivian Petersen Wagner:** validation, visualization, writing – review and editing. **Isabel Schausltz Pereira Faustino:** methodology, validation, visualization, writing – review and editing. **Marco Meleti:** validation, visualization, writing – review and editing. **Nikolaos Nikitakis:** validation, visualization, writing – review and editing. **Márcio Ajudarte Lopes:** validation, visualization, writing – review and editing. **Alan Roger Santos‐Silva:** visualization, writing – review and editing, validation, supervision, conceptualization. **Caique Mariano Pedroso:** conceptualization, validation, visualization, writing – review and editing, supervision, data curation, methodology.

## Funding

The authors have nothing to report.

## Ethics Statement

The authors have nothing to report.

## Conflicts of Interest

The authors declare no conflicts of interest.

## Supporting information


**Figure S1:** Flowchart evidencing the study selection process.

**Table S1:** Search strategy.

**Table S2:** Number of studies, countries, type of studies, diagnosis, clinical, and epidemiological variables from included studies for data extraction.

**Table S3:** Information about the treatments used in the excision of leukoplakia and proliferative verrucous leukoplakia, considering the total number of lesions treated and recurrences.

## Data Availability

The datasets generated during the current study are available from the corresponding author on reasonable request.
