# Supplementary material for: Management of Surgical Margins in the Excision of Oral Leukoplakia: A Scoping Review
Source: Oral Dis. 2026 Feb 13;32(7):1748–55. doi: 10.1111/odi.70231 (PMC13590898; doi:10.1111/odi.70231)
Supplement: Supplementary file 1 [file ODI-32-1748-s001.docx]

**Supplementary Table 1.** Search strategy.

| **Database** | **Search strategy**  **(search date: December 27^th^, 2024)** | **Results** |
| --- | --- | --- |
| **Pubmed** | (Leukoplakia[MeSH Terms] OR Leukoplakia[MeSH Subheading] OR Leukoplakia OR Leukoplakias OR "Leukoplakic Lesions" OR "Lesion, Leukoplakic" OR "Lesions, Leukoplakic" OR "Leukoplakic Lesion" OR Leukokeratosis OR Leukokeratoses OR "Leukoplakia, Oral" OR "Leukoplakias, Oral" OR "Oral Leukoplakia" OR "Oral Leukoplakias" OR "Leukokeratosis, Oral" OR "Leukokeratoses, Oral" OR "Oral Leukokeratoses" OR "Oral Leukokeratosis" OR "Keratosis, Oral" OR "Keratoses, Oral" OR "Oral Keratoses" OR "Oral Keratosis") AND (Therapeutics[MeSH Terms] OR Therapeutics[MeSH Subheading] OR Therapeutics OR Therapeutic OR Therapy OR Therapies OR Treatment OR Treatments)) AND ("Laser Therapy"[MeSH Terms] OR "Laser Therapy"[MeSH Subheading] OR "Laser Therapy" OR "Laser Therapies" OR "Therapies, Laser" OR "Therapy, Laser" OR "Laser Scalpel" OR "Laser Scalpels" OR "Scalpel, Laser" OR "Scalpels, Laser" OR "Laser Knives" OR "Knive, Laser" OR "Knives, Laser" OR "Laser Knive" OR "Laser Knife" OR "Knife, Laser" OR "Knifes, Laser" OR "Laser Knifes" OR "Laser Surgery" OR "Laser Surgeries" OR "Surgeries, Laser" OR "Surgery, Laser" OR "Vaporization, Laser" OR "Laser Vaporization" OR "Pulsed Laser Tissue Ablation" OR "Laser Photoablation of Tissue" OR "Laser Tissue Ablation" OR "Ablation, Laser Tissue" OR "Tissue Ablation, Laser" OR "Laser Ablation" OR "Ablation, Laser" OR "Nonablative Laser Treatment" OR "Laser Treatment, Nonablative" OR "Laser Treatments, Nonablative" OR "Nonablative Laser Treatments" OR "Dioxide Laser" OR "Carbon Dioxide Laser" OR "Laser Vaporization" OR Electrocoagulation OR Electrocautery OR Cryotherapies). | **341** |
| **Scopus** | TITLE-ABS-KEY ( leukoplakia OR leukoplakias OR "Leukoplakic Lesions" OR "Lesion, Leukoplakic" OR "Lesions, Leukoplakic" OR "Leukoplakic Lesion" OR leukokeratosis OR leukokeratoses OR "Leukoplakia, Oral" OR "Leukoplakias, Oral" OR "Oral Leukoplakia" OR "Oral Leukoplakias" OR "Leukokeratosis, Oral" OR "Leukokeratoses, Oral" OR "Oral Leukokeratoses" OR "Oral Leukokeratosis" OR "Keratosis, Oral" OR "Keratoses, Oral" OR "Oral Keratoses" OR "Oral Keratosis" ) AND TITLE-ABS-KEY ( therapeutics OR therapeutic OR therapy OR therapies OR treatment OR treatments ) AND TITLE-ABS-KEY ( "Laser Therapy" OR "Laser Therapies" OR "Therapies, Laser" OR "Therapy, Laser" OR "Laser Scalpel" OR "Laser Scalpels" OR "Scalpel, Laser" OR "Scalpels, Laser" OR "Laser Knives" OR "Knive, Laser" OR "Knives, Laser" OR "Laser Knive" OR "Laser Knife" OR "Knife, Laser" OR "Knifes, Laser" OR "Laser Knifes" OR "Laser Surgery" OR "Laser Surgeries" OR "Surgeries, Laser" OR "Surgery, Laser" OR "Vaporization, Laser" OR "Laser Vaporization" OR "Pulsed Laser Tissue Ablation" OR "Laser Photoablation of Tissue" OR "Laser Tissue Ablation" OR "Ablation, Laser Tissue" OR "Tissue Ablation, Laser" OR "Laser Ablation" OR "Ablation, Laser" OR "Nonablative Laser Treatment" OR "Laser Treatment, Nonablative" OR "Laser Treatments, Nonablative" OR "Nonablative Laser Treatments" OR "Dioxide Laser" OR "Carbon Dioxide Laser" OR "Laser Vaporization" OR electrocoagulation OR electrocautery OR cryotherapies ). | **445** |
| **Embase** | ('leukoplakia'/de OR leukoplakias OR 'leukoplakic lesions' OR 'lesion, leukoplakic' OR 'lesions, leukoplakic' OR 'leukoplakic lesion'/de OR 'leukokeratosis'/de OR 'leukokeratoses'/de OR 'leukoplakia, oral'/de OR 'leukoplakias, oral' OR 'oral leukoplakia'/de OR 'oral leukoplakias' OR 'leukokeratosis, oral' OR 'leukokeratoses, oral' OR 'oral leukokeratoses' OR 'oral leukokeratosis' OR 'keratosis, oral' OR 'keratoses, oral' OR 'oral keratoses' OR 'oral keratosis') AND ('therapeutics'/de OR therapeutic OR 'therapy'/de OR therapies OR 'treatment'/de OR treatments) AND ('laser therapy'/de OR 'laser therapies' OR 'therapies, laser' OR 'therapy, laser' OR 'laser scalpel'/de OR 'laser scalpels' OR 'scalpel, laser' OR 'scalpels, laser' OR 'laser knives' OR 'knive, laser' OR 'knives, laser' OR 'laser knive' OR 'laser knife'/de OR 'knife, laser' OR 'knifes, laser' OR 'laser knifes' OR 'laser surgery'/de OR 'laser surgeries' OR 'surgeries, laser' OR 'surgery, laser'/de OR 'vaporization, laser' OR 'pulsed laser tissue ablation' OR 'laser photoablation of tissue' OR 'laser tissue ablation' OR 'ablation, laser tissue' OR 'tissue ablation, laser' OR 'laser ablation'/de OR 'ablation, laser' OR 'nonablative laser treatment' OR 'laser treatment, nonablative' OR 'laser treatments, nonablative' OR 'nonablative laser treatments' OR 'dioxide laser' OR 'carbon dioxide laser'/de OR 'laser vaporization'/de OR 'electrocoagulation'/de OR 'electrocautery'/de OR cryotherapies). | **61** |
| **Web of Science** | **Leukoplakia OR Leukoplakias OR "Leukoplakic Lesions" OR "Lesion, Leukoplakic" OR "Lesions, Leukoplakic" OR "Leukoplakic Lesion" OR Leukokeratosis OR Leukokeratoses OR "Leukoplakia, Oral" OR "Leukoplakias, Oral" OR "Oral Leukoplakia" OR "Oral Leukoplakias" OR "Leukokeratosis, Oral" OR "Leukokeratoses, Oral" OR "Oral Leukokeratoses" OR "Oral Leukokeratosis" OR "Keratosis, Oral" OR "Keratoses, Oral" OR "Oral Keratoses" OR "Oral Keratosis"** (Topic) and **Therapeutics OR Therapeutic OR Therapy OR Therapies OR Treatment OR Treatments** (Topic) and **"Laser Therapy" OR "Laser Therapies" OR "Therapies, Laser" OR "Therapy, Laser" OR "Laser Scalpel" OR "Laser Scalpels" OR "Scalpel, Laser" OR "Scalpels, Laser" OR "Laser Knives" OR "Knive, Laser" OR "Knives, Laser" OR "Laser Knive" OR "Laser Knife" OR "Knife, Laser" OR "Knifes, Laser" OR "Laser Knifes" OR "Laser Surgery" OR "Laser Surgeries" OR "Surgeries, Laser" OR "Surgery, Laser" OR "Vaporization, Laser" OR "Laser Vaporization" OR "Pulsed Laser Tissue Ablation" OR "Laser Photoablation of Tissue" OR "Laser Tissue Ablation" OR "Ablation, Laser Tissue" OR "Tissue Ablation, Laser" OR "Laser Ablation" OR "Ablation, Laser" OR "Nonablative Laser Treatment" OR "Laser Treatment, Nonablative" OR "Laser Treatments, Nonablative" OR "Nonablative Laser Treatments" OR "Dioxide Laser" OR "Carbon Dioxide Laser" OR "Laser Vaporization" OR Electrocoagulation OR Electrocautery OR Cryotherapies** (Topic). | **157** |
| **Cochrane Library** | Leukoplakia OR Leukoplakias OR "Leukoplakic Lesions" OR "Lesion, Leukoplakic" OR "Lesions, Leukoplakic" OR "Leukoplakic Lesion" OR Leukokeratosis OR Leukokeratoses OR "Leukoplakia, Oral" OR "Leukoplakias, Oral" OR "Oral Leukoplakia" OR "Oral Leukoplakias" OR "Leukokeratosis, Oral" OR "Leukokeratoses, Oral" OR "Oral Leukokeratoses" OR "Oral Leukokeratosis" OR "Keratosis, Oral" OR "Keratoses, Oral" OR "Oral Keratoses" OR "Oral Keratosis" in Title Abstract Keyword AND Therapeutics OR Therapeutic OR Therapy OR Therapies OR Treatment OR Treatments in Title Abstract Keyword AND "Laser Therapy" OR "Laser Therapies" OR "Therapies, Laser" OR "Therapy, Laser" OR "Laser Scalpel" OR "Laser Scalpels" OR "Scalpel, Laser" OR "Scalpels, Laser" OR "Laser Knives" OR "Knive, Laser" OR "Knives, Laser" OR "Laser Knive" OR "Laser Knife" OR "Knife, Laser" OR "Knifes, Laser" OR "Laser Knifes" OR "Laser Surgery" OR "Laser Surgeries" OR "Surgeries, Laser" OR "Surgery, Laser" OR "Vaporization, Laser" OR "Laser Vaporization" OR "Pulsed Laser Tissue Ablation" OR "Laser Photoablation of Tissue" OR "Laser Tissue Ablation" OR "Ablation, Laser Tissue" OR "Tissue Ablation, Laser" OR "Laser Ablation" OR "Ablation, Laser" OR "Nonablative Laser Treatment" OR "Laser Treatment, Nonablative" OR "Laser Treatments, Nonablative" OR "Nonablative Laser Treatments" OR "Dioxide Laser" OR "Carbon Dioxide Laser" OR "Laser Vaporization" OR Electrocoagulation OR Electrocautery OR Cryotherapies. | **26** |
| **Google Scholar** | Leukoplakia OR Leukoplakias OR "Leukoplakic Lesions" OR "Lesion, Leukoplakic" OR "Lesions, Leukoplakic" OR "Leukoplakic Lesion" OR Leukokeratosis OR Leukokeratoses OR "Leukoplakia, Oral" OR "Leukoplakias, Oral" OR "Oral Leukoplakia" OR "Oral Leukoplakias" OR "Leukokeratosis, Oral" OR "Leukokeratoses, Oral" OR "Oral Leukokeratoses" OR "Oral Leukokeratosis" OR "Keratosis, Oral" OR "Keratoses, Oral" OR "Oral Keratoses" OR "Oral Keratosis" AND Therapeutics OR Therapeutic OR Therapy OR Therapies OR Treatment OR Treatments AND "Laser Therapy" OR "Laser Therapies" OR "Therapies, Laser" OR "Therapy, Laser" OR "Laser Scalpel" OR "Laser Scalpels" OR "Scalpel, Laser" OR "Scalpels, Laser" OR "Laser Knives" OR "Knive, Laser" OR "Knives, Laser" OR "Laser Knive" OR "Laser Knife" OR "Knife, Laser" OR "Knifes, Laser" OR "Laser Knifes" OR "Laser Surgery" OR "Laser Surgeries" OR "Surgeries, Laser" OR "Surgery, Laser" OR "Vaporization, Laser" OR "Laser Vaporization" OR "Pulsed Laser Tissue Ablation" OR "Laser Photoablation of Tissue" OR "Laser Tissue Ablation" OR "Ablation, Laser Tissue" OR "Tissue Ablation, Laser" OR "Laser Ablation" OR "Ablation, Laser" OR "Nonablative Laser Treatment" OR "Laser Treatment, Nonablative" OR "Laser Treatments, Nonablative" OR "Nonablative Laser Treatments" OR "Dioxide Laser" OR "Carbon Dioxide Laser" OR "Laser Vaporization" OR Electrocoagulation OR Electrocautery OR Cryotherapies. | **100** |
| **Proquest** | TI,AB(Leukoplakia OR Leukoplakias OR "Leukoplakic Lesions" OR "Lesion, Leukoplakic" OR "Lesions, Leukoplakic" OR "Leukoplakic Lesion" OR Leukokeratosis OR Leukokeratoses OR "Leukoplakia, Oral" OR "Leukoplakias, Oral" OR "Oral Leukoplakia" OR "Oral Leukoplakias" OR "Leukokeratosis, Oral" OR "Leukokeratoses, Oral" OR "Oral Leukokeratoses" OR "Oral Leukokeratosis" OR "Keratosis, Oral" OR "Keratoses, Oral" OR "Oral Keratoses" OR "Oral Keratosis") AND TI,AB(Therapeutics OR Therapeutic OR Therapy OR Therapies OR Treatment OR Treatments) AND TI,AB("Laser Therapy" OR "Laser Therapies" OR "Therapies, Laser" OR "Therapy, Laser" OR "Laser Scalpel" OR "Laser Scalpels" OR "Scalpel, Laser" OR "Scalpels, Laser" OR "Laser Knives" OR "Knive, Laser" OR "Knives, Laser" OR "Laser Knive" OR "Laser Knife" OR "Knife, Laser" OR "Knifes, Laser" OR "Laser Knifes" OR "Laser Surgery" OR "Laser Surgeries" OR "Surgeries, Laser" OR "Surgery, Laser" OR "Vaporization, Laser" OR "Laser Vaporization" OR "Pulsed Laser Tissue Ablation" OR "Laser Photoablation of Tissue" OR "Laser Tissue Ablation" OR "Ablation, Laser Tissue" OR "Tissue Ablation, Laser" OR "Laser Ablation" OR "Ablation, Laser" OR "Nonablative Laser Treatment" OR "Laser Treatment, Nonablative" OR "Laser Treatments, Nonablative" OR "Nonablative Laser Treatments" OR "Dioxide Laser" OR "Carbon Dioxide Laser" OR "Laser Vaporization" OR Electrocoagulation OR Electrocautery OR Cryotherapies). | **52** |
| **TOTAL** |  | **1,182** |
