# Supplementary material for: Management of Surgical Margins in the Excision of Oral Leukoplakia: A Scoping Review
Source: Oral Dis. 2026 Feb 13;32(7):1748–55. doi: 10.1111/odi.70231 (PMC13590898; doi:10.1111/odi.70231)
Supplement: Supplementary file 2 [file ODI-32-1748-s003.docx]

**Supplementary Table 2.** Number of studies, countries, type of studies, diagnosis, clinical and epidemiological variables from included studies for data extraction.

| **Variable** | **N** | **%** |
| --- | --- | --- |
| **Studies** | 91 | 100% |
| **Country** |  |  |
| India | 12 | 13.1% |
| Italy | 10 | 10.9% |
| Taiwan | 9 | 9.8% |
| USA | 7 | 7.6% |
| Germany | 6 | 6.5% |
| The Netherlands | 6 | 6.5% |
| Spain | 5 | 5.4% |
| United Kingdom | 5 | 5.4% |
| Brazil | 4 | 4.3% |
| Japan | 4 | 4.3% |
| China | 3 | 3.2% |
| England | 3 | 3.2% |
| Australia | 2 | 2.1% |
| Croatia | 2 | 2.1% |
| Poland | 2 | 2.1% |
| Russia | 2 | 2.1% |
| Belgium | 1 | 1% |
| Bulgaria | 1 | 1% |
| Czech | 1 | 1% |
| Denmark | 1 | 1% |
| France | 1 | 1% |
| Hungary | 1 | 1% |
| Nepal | 1 | 1% |
| Portugal | 1 | 1% |
| Turkey | 1 | 1% |
| **Type of study** |  |  |
| Retrospective study | 53 | 58.2% |
| Prospective study | 14 | 15.3% |
| Comparative study | 9 | 9.8% |
| Randomized controlled trial | 5 | 5.4% |
| Observational study | 4 | 4.3% |
| Longitudinal comparative clinical study | 1 | 1% |
| Multicentrical retrospective study | 1 | 1% |
| Prospective randomized study | 1 | 1% |
| Randomized, single-blinded, prospective parallel-group study | 1 | 1% |
| Retrospective and prospective study | 1 | 1% |
| Randomized controlled study | 1 | 1% |
| **Clinical diagnosis** |  |  |
| Oral leukoplakia | 9.082 | 98.2% |
| Proliferative verrucous leukoplakia | 163 | 1.76% |
| **Sex** |  |  |
| Male | 3.128 | 33.9% |
| Female | 1.751 | 18.9% |
| NR | 4.366 | 47.2% |
| **Alcohol consumption** |  |  |
| Yes | 534 | 5.8% |
| NR | 8.711 | 94.2% |
| **Tobacco consumption** |  |  |
| Yes | 1.244 | 13.5% |
| NR | 8.001 | 86.5% |
| **Age** |  |  |
| Mean | 57.2 | |
| Median | 58 | |
| Standard deviation | 3.18 | |
| Minimum | 53 | |
| Maximum | 63 | |
| NR | 48 (studies) | |
| **Follow up time (months)** |  | |
| Mean | 29.4 | |
| Median | 24 | |
| Standard deviation | 22.5 | |
| Minimum | 0 | |
| Maximum | 80 | |
| NR | 26 (studies) | |

NR: Not reported.
