# Supplementary material for: Management of Surgical Margins in the Excision of Oral Leukoplakia: A Scoping Review
Source: Oral Dis. 2026 Feb 13;32(7):1748–55. doi: 10.1111/odi.70231 (PMC13590898; doi:10.1111/odi.70231)
Supplement: Supplementary file 3 [file ODI-32-1748-s004.docx]

**Supplementary Table 3.** Informations about the treatments used in the excision of leukoplakia and proliferative verrucous leukoplakia, considering the total number of lesions treated and recurrences.

| **Variable** | | **OL**  **N treated / (%)** | | **OL**  **N recurrence / (%)** | | **PVL**  **N treated / (%)** | | **PVL**  **N recurrence / (%)** |
| --- | --- | --- | --- | --- | --- | --- | --- | --- |
| **Type of treatment used in excision** | |  | |  | |  | |  |
| Argon Plasma Coagulator | | 12 / (0.13%) | | 0 / (0%) | | - | | - |
| CO2 Laser | | 6.715 / (73.9%) | | 703 / (10.4%) | | 117 / (71.7%) | | 33 / (28.2%) |
| CO2 Laser and Diode Laser | | 49 / (0.53%) | | 15 / (30.6%) | | - | | - |
| CO2 Laser and Nd:YAG Laser | | 48 / (0.52%) | | 1 / (2.08%) | | 14 / (8.58%) | | 1 / (7.14%) |
| CO2 Laser and Traditional Scalpel | | 180 / (1.98%) | | 26 / (14.4%) | | - | | - |
| Cryosurgery | | 451 / (4.96%) | | 68 / (15%) | | - | | - |
| Diode Laser | | 333 / (3.66%) | | 31 / (9.3%) | | 8 / (4.9%) | | NR |
| Er,Cr:YSGG Laser | | 27 / (0.29%) | | 0 / (0%) | | - | | - |
| Er:YAG Laser | | 355 / (3.90%) | | 55 / (15.4%) | | - | | - |
| Er:YAG Laser and CO2 Laser | | 16 / (0.17%) | | 2 / (12.5%) | | - | | - |
| Nd:YAG Laser | | 143 / (1.57%) | | 31 / (21.6%) | | - | | - |
| KTP Laser | | 30 / (0.33%) | | 7 / (23.3%) | | - | | - |
| QMR Lancet | | 8 / (0.08%) | | 2 / (25%) | | - | | - |
| Traditional Scalpel | | 715 / (7.87%) | | 89 / (12.4%) | | 24 / (14.7%) | | NR |
| Total | | 9.082 / (100%) | | 1.030 / (11.3%) | | 163 / (100%) | | 34 / (20.8%) |
| **How were the lesions treated?** |  | |  | |  | |  | |
| Ablation | | 645 / (7.1%) | | 129 / (20%) | | - | | - |
| Excision | | 3.180 / (35%) | | 457 / (14.3%) | | - | | - |
| Vaporization | | 957 / (10.5%) | | 196 / (20.4%) | | 19 / (11.6%) | | 3 / (15.7%) |
| Excision combinate with vaporization/ ablation/ coagulation | | 3.792 / (41.7%) | | 180 (4.7%) | | 102 / (62.5%) | | 31 / (30.3%) |
| Photocoagulation | | 72 / (0.79%) | | 0 / (0%) | | - | | - |
| Frozen | | 427 / (4.70%) | | 68 / (15.9%) | | - | | - |
| NR | | 0 / (0%) | | - | | 42 / (25.7%) | | - |

OL: Oral leukoplakia; PVL: Proliferative verrucous leukoplakia; NR: Not reported; CO2: Carbon dioxide; Nd:YAG: Neodymium-doped yttrium aluminium garnet; Er,Cr:YSGG: Erbium, chromium: yttrium scandium gallium garn; Er:YAG: Erbium-doped yttrium aluminum garnet; KTP: Potássio-titanil-fosfato; QMR: Quantic molecular resonance.
